# A Blessing in Disguise: Flanking Words Can Cancel Language Switch Costs

**DOI:** 10.5334/joc.332

**Published:** 2024-01-30

**Authors:** Aaron Vandendaele, Nicoleta Prutean, Mathieu Declerck

**Affiliations:** 1 Ghent University, Ghent, Belgium; 2 Vrije Universiteit Brussel, Brussels, Belgium

**Keywords:** Bilingual, Comprehension, Language control, Language switching, Flankers task

## Abstract

Prior research has shown that a sentence context can decrease the necessity for language control relative to single word processing. In particular, measures of language control such as language switch costs are reduced or even absent in a sentence context. Yet, this evidence is mainly based on bilingual language production and is far from straightforward. To further investigate this issue in the comprehension modality, we relied on the lexical flanker task, which is known to introduce sentence-like processing. More specifically, Dutch-English bilinguals (n = 68) performed a classification task in mixed language blocks on target words that were either presented alone or flanked by unrelated words in the same language. While overall no L1 switch costs were observed, we only observed L2 switch costs in the no-flanker condition. This pattern of results indicates that the presence of flankers can reduce or even abolish switch costs, suggesting that the language control process can benefit from sentence(-like) processing compared to single word processing.

## Introduction

Language control refers to the cognitive mechanism that enables word selection in a target language by reducing cross-language interference from the non-target language (e.g., Declerck & Koch, 2023; Green, 1998). Recent research on this topic (e.g., Blanco-Elorrieta & Pylkkänen, 2018; Peeters & Dijkstra, 2018; Sánchez et al., 2021) has emphasized the importance of investigating language control processes in situations that closely resemble every-day language use, such as language control during sentence processing instead of single word processing (e.g., Declerck et al., 2021; Li & Gollan, 2021). However, existing research does not provide an entirely clear understanding of how (or even if) language control is affected by sentence processing relative to single word processing. To investigate the influence of a sentence-like context, relative to a single word context, on language control, we implemented a flanker paradigm based on the established work of Jonathan Grainger (e.g., Meade et al., 2021; Vandendaele & Grainger, 2022).

One line of evidence that sentences can affect language control is that several production studies found no evidence for language control when relying on language switching in a sentence context (e.g., Bultena et al., 2015; Gullifer et al., 2013). For instance, Gullifer et al. (2013) let English-Spanish bilinguals silently read a written sentence, apart from one marked word that was produced out loud. After every second sentence, the language of the sentence changed. The results showed no performance costs for the marked word between sentences that were written in a different language from the previous sentence relative to sentences that were written in the same language as the previous sentence. This is surprising, as single word production studies that rely on a similar language alternating setup tend to show a performance cost (e.g., Declerck et al., 2015; Jackson et al., 2001; Wong & Maurer, 2021), with worse performance after switching to another language relative to language repetition (i.e., switch costs). Since these switch costs are taken as a measure of language control (e.g., Declerck & Philipp, 2015a; Green, 1998), it could be deduced that little to no language control might have occurred in the study of Gullifer et al. (2013).

A decrease or even absence of language control during sentence production compared to single words is often attributed to lower cross-language interference, as suggested by the cognate facilitation effect (Declerck et al., 2021., Li & Gollan, 2021). This effect –often used as a measure of cross-language interference– shows improved performance for translation-equivalent words that sound and/or look highly similar across languages compared to translation-equivalent words that sound dissimilar (e.g., an advantage for ‘*good*’ and ‘*goed*’ compared to ‘*eye*’ and ‘*oog*’ for an English-Dutch bilingual, Costa et al., 2000; Hoshino & Kroll, 2008). Interestingly, a reduction of this effect has been observed in a sentence context relative to single words production, especially when the sentences have a high semantic constraint (e.g., Schwartz et al., 2008; Schwartz & Kroll, 2006; Starreveld et al., 2014). These results of decreased cognate facilitation can be interpreted as less cross-language interference being needed in a sentence context.

Though, contrary to Gullifer et al. (2013), several language switching studies that focused on bilingual sentence production observed switch costs (e.g., Declerck et al., 2017, 2021; Tarlowski et al., 2013). For instance, Declerck et al. (2017) relied on a network description task during which French-English bilinguals had to describe the route a dot made over a network of different lines (i.e., straight, curved, and diagonal lines) that connected different pictures. Each stage of the network required the description of the direction, type of line, and picture in a sentence in either of their two languages, depending on the visual language cues (i.e., different colored frames around each picture). The results showed that more language intrusions (involuntary usage of the non-target language) were produced during switch trials than during repetition trials. This switch cost effect has been replicated in two subsequent studies that used a similar network description task but relied on different bilinguals and different dependent variables (Declerck et al., 2021; Sánchez et al., 2021). Together these results indicate that switch costs can be observed during sentence production, and accordingly that some language control can be needed in a sentence context.

Thus far, the described studies have focused on language control within a sentence context. However, there has been little research directly comparing language switching in a sentence context with that in a single word context. The few studies that did investigate this issue relied on within-sentence language switching (Declerck & Philipp, 2015b; Li & Gollan, 2021). In Declerck and Philipp (2015b), German-English bilinguals had to memorize five words that could either be in a sentence sequence (e.g., *this boy runs very fast*) or in a scrambled, non-sentence sequence (e.g., *runs boy fast very this*), while alternating languages after every second word in the sequence. Their results showed switch costs in the latter condition, but no significant switch costs were observed when the five-word sequence comprised a grammatically correct sentence in both languages (language-unspecific sentences). When the five-word sequence comprised a grammatically correct sentence in one language but not the other (language-specific sentences), a comparable switch cost pattern was observed as in the scrambled, non-sentence condition. Hence, it seems that larger switch costs, and thus more language control, can occur in a non-sentence setting relative to a (language-unspecific) sentence setting.

A different approach was taken by Li and Gollan (2021), who compared language switching performance during picture naming and during picture naming in a written sentence context. In contrast to Declerck and Philipp (2015b), their results showed larger switch costs in a sentence context than during single word production. However, the setup of Li and Gollan allowed their participants to adopt a default language (i.e., the language of the written sentence), which is known to increase switch costs if switched out of (Gollan & Goldrick, 2016). No default language would be adopted in Declerck and Philipp (2015b) because either language was used about 50% of the time in each sentence.

This overview provides some evidence suggesting that language control is affected by a sentence context, but the evidence is scarce and far from straightforward. Moreover, these studies focused mostly on language production, whereas the issue in language comprehension remains largely unexplored and worthwhile to investigate, given that production- and comprehension-based language control are not identical (e.g., Blanco-Elorrieta & Pylkkänen, 2016; Mosca & de Bot, 2017; however, see Peeters et al., 2014).

To the best of our knowledge, no direct evidence has shown that comprehension-based language control is affected by a sentence context relative to a single word context. Furthermore, switch costs in bilingual language comprehension are generally less robust than in bilingual language production (for a discussion, see Declerck et al., 2019). Consequently, relying on the presence or absence of switch costs in sentence processing studies (e.g., Olson, 2016; Philipp & Huestegge, 2015) to compare them with comprehension-based language switch costs during single word processing (Declerck & Grainger, 2017; Struck & Jiang, 2021) is insufficient to make a claim about whether comprehension-based language control is affected by a sentence context.

### Current study

So, unlike prior studies investigating language control in a sentence context vs. single word context, we focused on bilingual language comprehension. Another difference from the current literature on this topic, is that we focused solely on one aspect that differs between a sentence and single word context. Sentences and single words differ in terms of aspects such as the depth of grammatical processing and the influence of possible adjacent words.

In order to directly investigate how language control affects the influence of possible adjacent words in sentence context vs. single word context, we relied on the presence of flankers (or lack thereof) to have comparable sentence and single word conditions. Since the flanking words are not grammatically connected to the target word, as all words are semantically-unrelated nouns, this aspect should not further constrain word selection. Yet, the presence of adjacent words could have an impact on processing the target word.

Recent research has shown that language comprehension of a word that is flanked by unrelated words can be processed in a similar fashion compared to a sentence context (Meade et al., 2021; Vandendaele & Grainger, 2022). In the reading version of the flankers paradigm, participants have to perform a task on the middle word (e.g., semantic categorization task), whilst ignoring the unrelated flanker words. Important to note is that regardless of the actual task, performance was always compared between a flanker and a no-flanker condition. For instance, Meade et al. (2021) investigated neighborhood density (i.e., the number of words that have the same length but differ regarding one letter) with and without unrelated flankers. Generally, increasing neighborhood density of a word facilitates performance during single word processing and inhibits during sentence processing. Along the claim that unrelated flankers result in sentence-like processing for the target word, performance was more inhibited with flankers. Further evidence comes from Vandendaele and Grainger (2022), who looked at the word concreteness effect (i.e., the finding that concrete words are processed faster and more accurate than abstract words) using the same flanker/no-flanker manipulation. The word concreteness effect is typically found in a sentence context, and as such, has been taken as evidence that words with high concreteness ratings elicit more semantic processing. In line with this hypothesis, word concreteness effects were only found in the presence of unrelated flankers, while no such effect was observed in the no flanker condition. These two studies suggest that the mere presence of unrelated flankers is enough to encourage minimal sentence(-like) processing.

Therefore, a similar flanker/no-flanker setup as in Meade et al. (2021) and Vandendaele and Grainger (2022) was used in the current study. More specifically, Dutch-English bilinguals performed a size categorization task on target words (i.e., does the target word represent an object that is larger or smaller than 1 meter?) presented with unrelated flankers (in the same language as the target word) in one block and without flankers in the other block.

According to the BIA (Grainger & Dijkstra, 1992; van Heuven et al., 1998) and BIA-d (Grainger et al., 2010) models, processing of a word representation (e.g., cat) automatically activates the corresponding language node (i.e., the English node in the example of cat), which is a mental representation of language membership. Subsequently, the language node inhibits all word representations of the other language. Hence, if language membership of adjacent words does not influence word processing, we would not expect any switch cost difference between the flanker and no-flanker condition according to the models we just described. If language membership of adjacent words does have an impact on word processing, there are two possible outcomes according to the models discussed above. (1) It could be that the increase in language activation in the previous trial, due to the flankers, increases the inhibition of the target language in switch trials (cf. proportional language control; Green, 1998). In turn, more inhibition needs to be overcome in switch trials and thus there should be an increase in switch costs in the flanker condition. (2) Another possibility is that the increase of language activation, due to the flankers, aids the necessity of overcoming inhibition on switch trials, and thus makes switch costs substantially smaller in the flanker condition.

## Methods

### Participants

**Figure 1:**
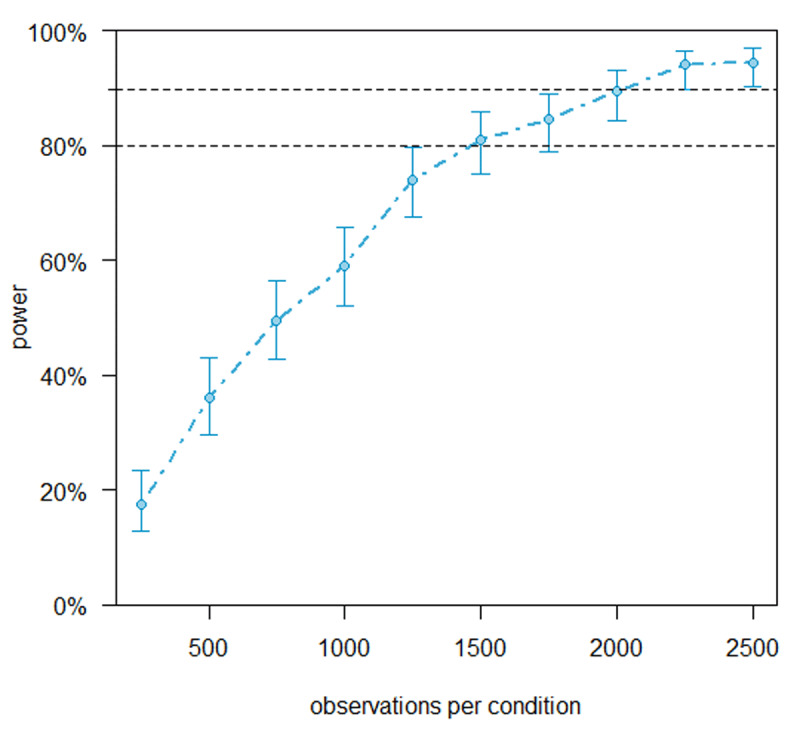
Power for the interaction effect between Concreteness and flanker presence at multiple levels of observations per condition.

A power analysis was performed on the data of Vandendaele and Grainger (2022) to determine the required number of participants. Because this study did not investigate switch costs, the Concreteness factor in Vandendaele and Grainger was used as a proxy for switch costs. Using the SimR package (Green and MacLeod, 2016) in RStudio, we performed 200 Monte Carlo simulations on a range of sample sizes. Power was simulated at increments of 250 observations per condition. Importantly, each simulation used a linear mixed model structure that comprised the critical interaction effect between Concreteness and Flanker presence. Results of these simulations can be seen in Figure 1. Our simulations showed that the 90% power threshold for the interaction effect (cf. Brysbaert, 2019) was reached starting from 2000 observations per condition. In our design, this translates into collecting the data of 68 Dutch-English bilinguals. Because we relied on the data of Vandendaele and Grainger (2022) for the power analysis, we kept the rest of the methodology as similar as possible to that study.

Prior to the experiment, the participants were asked to fill in a language background questionnaire that provided information about their age-of-acquisition (AoA), percentage of current language use, and self-rated scores for speaking and reading both Dutch and English. A scale from 1 (low) to 7 (high) was used for the self-rated scores (see Table 1). After the main experiment, participants were asked to complete a an English vocabulary test based on lexical decision tasks (i.e., LexTale; Lemhöfer & Broersma, 2012).

**Table 1:** Overview of the participant descriptives.


	ENGLISH	DUTCH

Percentage used	39.6 (27.3)	58.8 (27.5)

Reading fluency	6.6 (0.6)	6.9 (0.3)

Speaking fluency	6.1 (0.9)	6.6 (0.6)

Writing fluency	5.9 (1.0)	6.4 (0.7)

AoA	10.6 (2.7)	N.A.

LexTale	87.3 (14.3)	N.A.


*Note*: Values between parentheses indicate standard deviations.

### Stimuli

The stimuli consisted of 60 English words, half of which represented an object that is smaller than one meter and the other half represent an object that is larger than one meter. Furthermore, we also included 60 target Dutch words, which were the translation-equivalent of the target English words (for an overview of the stimuli properties, see Table 2). None of these words were identical cognates, as some comprehension studies have shown that switch costs can be affected by cognate status (Thomas & Allport, 2000). The average orthographic Levenshtein distance between the translation-equivalent targets was 4.17 with a standard deviation of 1.34.

**Table 2:** Overview of the stimuli properties.


	ENGLISH	DUTCH

Target Frequency^1^	4.30 (0.47)^a^	2.72 (0.96)^b^

Flanker Frequency	4.78 (0.38)^a^	3.51 (0.58)^b^

Target & Flanker Length	5.00 (0.82)	5.03 (1.15)

Target Concreteness	4.77 (0.28)^c^	4.60 (0.46)^d^

Flanker Concreteness	2.88 (0.92)^c^	2.36 (0.75)^d^


*Note*: Values between parentheses indicate standard deviations. ^a^Subtlex-UK (van Heuven et al., 2014). ^b^Subtlex-NL (Keuleers et al., 2010). ^c^Brysbaert et al., 2014a. ^d^Brysbaert et al., 2014b.

For every target word there was also a flanker word, which was semantically unrelated to the target word, from the same language as the target word, and had the same length in terms of number of letters. Compared to the target word, flankers in both languages were of a higher frequency to promote processing (e.g., Segui & Grainger, 1990). Furthermore, each of these flanker words were also non-cognates, but unlike the target words, they were unrelated to size (e.g., abstract words, such as *dream*) to prevent any congruent or incongruent task responses relative to the target words (for a full overview of all target and distractor words, see Appendix).

### Apparatus

The experiment was designed using OpenSesame (Mathôt et al., 2012) and presented online with the the OSWeb extension into JATOS (Lange et al., 2015). Participants used their own personal computer to complete the experiment. The stimuli were presented using a 30-point monospaced font (droid sans mono) in lowercase.

### Procedure

The experiment consisted of two 60-trial blocks. In one of the blocks, only the target words were presented and in the other block each target word was flanked by their corresponding unrelated flanker word. The order of these blocks was counter-balanced across participants. Furthermore, every Dutch target word and its translation-equivalent English target word appeared once throughout the experiment. The occurrence of target words, and their translation-equivalent counterparts in the two block conditions were also counterbalanced across participants.

Prior to each block, participants were instructed to perform a size categorization task on the target word (i.e., is the object smaller or larger than 1 meter by pressing ‘g’ or ‘h’, respectively, on their azerty keyboard). Additionally, in the block that contained flankers, participants were instructed that each target word would be flanked on the left and right side by another word, but they should focus on the middle target word. These instructions were followed by a practice block of 12 trials, after which the actual experimental blocks were presented.

Each block had a random presentation of the target words. However, to have a similar number of trials in each language (i.e., Dutch and English) and with each language transition type (i.e., switch and repetition trials), a pseudorandomized sequence list was used. We created two different sequence lists in which the order of switch and non-switch trials was random but the amount of switch and non-switch trials was controlled for. After randomizing the sequence lists, we made sure that both lists did not have more than 3 subsequent trials in the same language.

Every trial started with the presentation of two vertically aligned fixation bars for 500 ms. This was followed by the stimuli, which stayed on the screen for a duration of 170 ms (cf. Declerck et al., 2018; Meade et al., 2021; Vandendaele & Grainger, 2022). Following the presentation of the stimuli, participants could respond for 2000 ms. Feedback was only given in the practice blocks for a random duration of 500–700 ms through a green or red dot following a correct or incorrect response, respectively.

### Analyses

The RT and error data was analyzed using frequentist (generalized) linear mixed-effects regression modeling (Baayen et al., 2008). All models were fitted with the lmer (RTs) or glmer (error rates) functions from the lme4 package (Bates et al., 2015). In addition, we also fitted Bayesian mixed-effects regression modeling using the brms package (Bürkner, 2017). For the frequentist approach, items and participants were entered as crossed random effects. Additionally, when the model structure allowed it, by-item and by-participant random slopes were included (Baayen et al., 2008; Barr et al., 2013). Lastly, RT data were log-transformed to assume a normal distribution. We report *b*-values, standard errors (*SE*s) and *t*- or *z*-values with the frequentist approach, with those beyond |1.96| deemed as significant. In the Bayesian approach, all models were fit with 3000 iterations for warm-up and 17000 iterations for sampling. Priors were set as obtained from the *get_prior* function in brms. These priors can be seen as weakly informative. The RT models were run under a lognormal distribution. For each factor, we report point & error estimations, the 95% credible interval, the Rhat convergence statistic and the number of effective sample size (ESS). Evidence for an effect was deemed meaningful if the 95% credibility interval of the posterior distribution did not include 0 (all analytical scripts and trial-level data can be found at https://osf.io/gu76e/).

We excluded two participants for scoring below chance level (2.9%). Regarding RT analyses, the first trial of each block, error trials, and trials following an error were excluded (17.8%). Moreover, RTs that exceeded 2.5 standard deviations from the grand mean were also discarded (2.8%). For the analyses of error rates, the first trial of each block and trials following an error were excluded (12.1%).

## Results

### Reaction times

**Figure 2:**
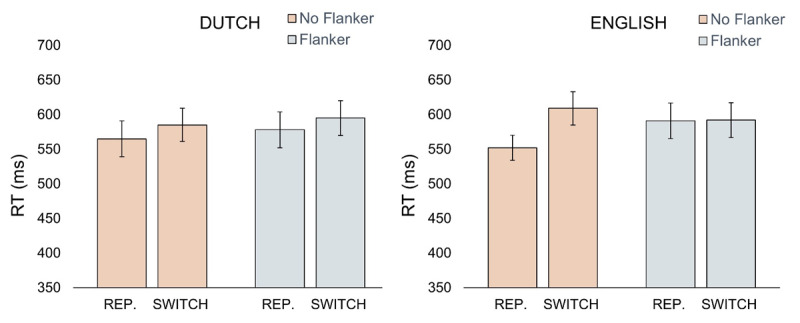
Descriptives for the RT data for all conditions. Error bars depict standard errors. Language refers to target at trial n, switch/repetition bars refers to the target language at trial n compared to the target language at trial n-1. REP. refer to repetition trials.

**Figure 3:**
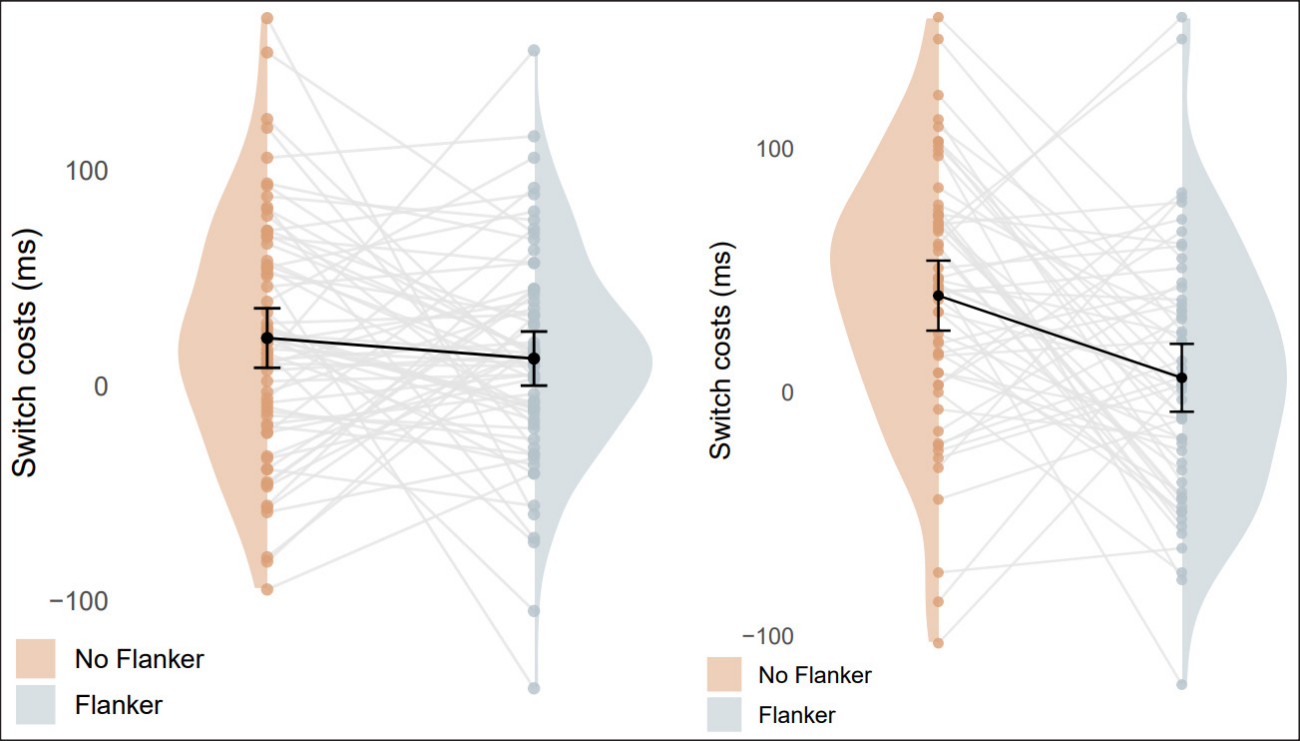
switch costs in RT plotted per participant with and without the presence of flankers. The figure on the left side depicts Dutch (L1) trials and on the right English (L2) trials. Switch costs are depicted as switch trials – repetition trials.

As can be seen in Table 3 (see also Figure 2 & 3), there was a significant effect of flanker presence (b = 1.31, SE = 0.55, t = 2.39), indicating that participants were slower when flankers were present. There was also a significant interaction between language and trial type (b = 1.55, SE = 0.68, t = 2.28), indicating significantly larger L2 switch costs (29 ms; b = 1.95, SE = 0.71, t = 2.77) compared to non-significant L1 switch costs (18 ms; b = 0.27, SE = 0.76, t = 0.36).

**Table 3:** Descriptives for RTs.


	DUTCH			ENGLISH		
	
	SWITCH	NO SWITCH	SWITCH COST	SWITCH	NO SWITCH	SWITCH COST

Flanker present	595 (208)	578 (200)	17	592 (191)	591 (196)	1

Flanker absent	585 (214)	565 (216)	20	609 (211)	552 (180)	57


*Note*: Values between parentheses indicate standard deviations.

Importantly, there was a significant three-way interaction (b = –2.04, SE = 0.96, t = –2.12), indicating no significant L1 switch costs both with (20 ms; b = 1.29, SE = 1.23, t = 1.00) and without (17 ms; b = 2.15, SE = 1.33, t = 1.62) the flankers, whereas L2 switch costs were not significant with the flankers (1 ms; b = 0.36, SE = 1.05, t = 0.34) but present without the flankers (57 ms; b = 4.18, SE = 1.16, t = 3.59). The rest of the effects were not significant (see Table 2). The Bayesian results were entirely in line with the linear mixed-effects regression modeling. For an overview of the analyses, see Table 4.

**Table 4:** LMM analyses for RTs.


FACTORS	FREQUENTIST LMM			BAYESIAN LMM						
	
	*B*-VALUE	SE	*T*-VALUE	*ESTIMATE*	*ERROR*	*LOWER BOUND*	*UPPER BOUND*	*^R*	*BULK ESS*	*TAIL ESS*

Flanker presence	1.31	0.55	**2.39**	0.022	0.009	**0.003**	**0.042**	1.00	20031	35401

Language	0.28	0.58	0.49	0.009	0.009	–0.008	0.026	1.00	20278	34405

Trial type	0.56	0.48	1.16	0.004	0.011	–0.017	0.024	1.00	22531	37757

Flanker × Language	0.29	0.83	0.36	0.011	0.012	–0.013	0.036	1.00	21094	35919

Flanker × Trial type	0.59	0.69	0.87	0.006	0.015	–0.023	0.036	1.00	18492	33965

Language × Trial type	1.55	0.79	**2.28**	0.030	0.012	**0.006**	**0.054**	1.00	21385	35332

Flanker × Language × Trial type	–2.04	0.96	**-2.12**	-0.037	0.017	**–0.071**	–**0.004**	1.00	20906	38016


### Error rates

We did not observe any significant effects in the error rates, nor any evidence for effects in the Bayesian analysis (see Tables 5 and 6).

**Table 5:** descriptives for error rates.


	DUTCH			ENGLISH		
	
	SWITCH	NO SWITCH	SWITCH COST	SWITCH	NO SWITCH	SWITCH COST

Flanker present	9.75 (0.70)	8.79 (0.72)	0.96	7.06 (0.74)	7.89 (0.73)	–0.83

Flanker absent	11.86 (0.68)	6.13 (0.76)	5.73	8.08 (0.73)	4.91 (0.78)	3.17


*Note*: Values between parentheses indicate standard deviations.

**Table 6:** LMM analyses for error rates.


FACTORS	FREQUENTIST LMM			BAYESIAN LMM						
	
	*B*-VALUE	SE	*Z*-VALUE	*ESTIMATE*	*ERROR*	*LOWER BOUND*	*UPPER BOUND*	*^R*	*BULK ESS*	*TAIL ESS*

Flanker presence	–0.17	0.21	–0.79	–0.15	0.20	–0.55	0.25	1.00	35493	46639

Language	0.23	0.21	1.06	0.24	0.20	–0.15	0.63	1.00	38165	47123

Trual type	–0.20	0.21	–0.95	–0.18	0.21	–0.58	0.22	1.00	38628	50024

Flanker × Language	–0.08	0.28	–0.29	–0.10	0.26	–0.61	0.41	1.00	35303	46924

Flanker × Trial type	0.19	0.30	0.62	0.15	0.28	–0.40	0.70	1.00	35862	46979

Language × Trial type	0.24	0.27	0.90	0.22	0.25	–0.27	0.71	1.00.	37387	48924

Flanker × Language × Trial type	–0.15	0.38	–0.39	–0.12	0.34	–0.77	0.55	100	37817	49371


## Discussion

To investigate the possible influence of adjacent words on comprehension-based language control, we contrasted categorization performance of target words with and without flankers. The results showed little to no L1 switch costs both with and without flankers. However, L2 switch costs were substantially diminished, to the point of being absent, in the condition with flankers relative to the condition without flankers.

The observation of smaller L2 switch costs with flankers offer evidence suggesting that adjacent words aid the language control process in the context of bilingual language comprehension. These reduced switch costs –which are a measure of language control (e.g., Declerck & Philipp, 2015a; Green, 1998)– are in line with the majority of production-based language control studies that have relied on a sentence context. For instance, it is in line with the language switching studies that observed no switch costs in a sentence context (e.g., Bultena et al., 2015; Gullifer et al., 2013), whereas a similar design during single word processing tends to lead to substantial switch costs (e.g., Declerck et al., 2015; Jackson et al., 2001; Wong & Maurer, 2021). It is also in line with the study of Declerck and Philipp (2015b), in which no switch costs were observed in language-unspecific sentences, whereas substantial switch costs were observed in scrambled sentences.

Our results are also in line with a relatively recent language comprehension study. In Declerck et al. (2020), participants were also presented with either a scrambled or correct four-word sequence. However, the key manipulation here was that the word sequences were mixed language (e.g., *ses feet sont big*). Each four-word sequence was presented for 200 ms, after which one word was post-cued for identification. Results showed a clear advantage for sentences that had a correct syntactic structure (i.e., a sentence superiority effect), further indicating that a mixed language context during sentence processing need not have a severe impact.

The decreased necessity for language control in a sentence context compared to a single word context has been explained by reduced cross-language interference in the context of sentences (e.g., Lauro & Schwartz, 2017; Libben & Titone, 2009; van Hell & de Groot, 2008), which in turn should lead to less language control to deal with said cross-language interference (e.g., Declerck et al., 2021). The lower cross-language interference in sentences could be explained by grammatical constraints that reduce the number of word representations that could be selected in a sentence. In turn, this will reduce the overall amount of cross-language interference, because less non-target words should reach a substantial level of activation in sentences.

Though this account, based on grammatical constraints, cannot be used to explain the current findings, as there was no clear grammatical connection between the target word and its flankers. However, prior research has indicated that when the target word is presented shortly with words on either side, this leads to sentence(-like) processing, compared to when the target word is presented without unrelated flankers (Meade et al., 2021; Vandendaele & Grainger, 2022). For instance, Vandendaele and Grainger (2022) showed a concreteness effect with flankers, even though this effect is typically only observed in the context of sentences. In the no flanker condition, no such effect was observed. The authors hypothesized that the key mechanism which triggers a more sentence-like processing is the visual presence of plausible word entities as flankers. Indeed, even when flankers were pseudo- or non-words, the effect of concreteness was still be observed. Hence, the visual presence of a sentence context seems crucial to provide a sentence-like context in written language processing. In contrast, other single word presentation paradigms like the Rapid Serial Visual Presentation (RSVP) still provide a sentence-like context but gives no opportunity for multiple words to be integrated spatially (i.e., Snell et al., 2018). Future research could examine whether a sentence context needs to be triggered by visually present lexical entities in order to affect language control processes in written word processing.

This still leaves the question of why smaller switch costs were observed with flankers than without. A possible explanation of the results, based on the BIA and BIA-d (Grainger & Dijkstra, 1992; Grainger et al., 2010; van Heuven et al., 1998), is that when bilinguals switch from Language A to Language B, adjacent words in the same language (i.e., Language B) increase the overall activation of this language because more words of Language B feed into the language node of Language B (cf. Grainger & Dijkstra, 1992; Grainger et al., 2010; van Heuven et al., 1998). In turn, it should be easier to overcome the inhibition of Language B, which persists from the previous trial to the current trial when switching languages, because the activation of Language B is increased by the flankers.

As we have indicated in the introduction, flanking words might also increase the inhibition of the non-target language in the BIA and BIA-d (Grainger & Dijkstra, 1992; Grainger et al., 2010; van Heuven et al., 1998), which should increase switch costs. However, we need to take into account that reactive, persisting inhibition should decrease over time. Otherwise it would continuously impact a bilinguals language processing, regardless of whether it is appropriate in the current context. So, even if inhibition of the non-target language increases due to flankers, it would probably not be as impactful as the influence of the currently present flankers and target word. Consequently, the increased language activation of current flankers should outweigh the persisting inhibition of previously presented flankers (in a language switch context).

The fact that we observed no L1 switch costs and substantial L2 switch costs also entails that we observed smaller L1 than L2 switch costs. While such a pattern is not always observed in the comprehension literature (e.g., Declerck & Grainger, 2017; Mosca & de Bot, 2017), it can be explained by the BIA and BIA-d models (Grainger & Dijkstra, 1992; Grainger et al., 2010; van Heuven et al., 1998): According to these models, reading a word will lead to activation of its word representation. In turn, its corresponding language node, which is a mental representation of a specific language, will also be activated. This language node will inhibit all words that are not part of that language. So, when another language is used on a consecutive trial, this inhibition will make it harder to select words in the target language, and thus lead to switch costs. Because the inhibition of the language node depends on the strength with which it is activated by the word, L1 will inhibit L2 more than vice versa. This occurs because L1 words typically have a larger base activation as they are used more in daily life. In turn, L2 will be inhibited more than L1 and thus when using L2 after L1, more inhibition will have to be overcome than vice versa. Consequently, this should result in larger L2 than L1 switch costs.

Taken together, the current study showed no overall L1 switch costs and larger L2 switch costs without than with unrelated flanker words. Since unrelated flanker words are known to elicit a sentence context (Meade et al., 2021; Vandendaele & Grainger, 2022), this latter finding could be interpreted in terms of language control benefiting from a sentence context relative to a single word context.

## Data Accessibility Statement

All raw data and analyses scripts can be accessed though the following link: https://osf.io/gu76e/.

## Footnotes

Note that the frequency ratings between languages are comparable, but the numbers differ because word frequency is measured logarithmically in the SUBTLEX-databases, for which the UK version has more listings.

## Appendix

### Word list

*Note*: Targets larger than 1 m are underlined, targets smaller than 1m are in *italics*.

**Table:**

ENGLISH		DUTCH	

TARGET	FLANKER	TARGET	FLANKER

army	feed	leger	kwaad

city	fuel	stad	niks

lion	duty	leeuw	spijt

cave	flow	grot	kijk

road	wise	weg	tof

barn	soul	schuur	gelijk

pool	fake	zwembad	rotzooi

lawn	riot	gazon	buurt

bull	whom	stier	flauw

gown	rush	gewaad	vlucht

plane	story	vliegtuig	waarschuw

tower	final	toren	dwaas

whale	music	walvis	eender

field	truth	veld	grap

crowd	state	volk	tijd

floor	taste	vloer	angst

cabin	power	hut	mal

hedge	trust	haag	kind

coast	every	kust	gaaf

vault	speed	kluis	zonde

garden	bottom	tuin	dank

church	damage	kerk	slim

bridge	common	brug	ziel

stairs	nobody	trap	ding

throne	liquid	troon	hekel

sailor	extent	zeiler	lokaas

shower	actual	douche	partij

galaxy	choice	heelal	strijd

shovel	spirit	schop	reden

convoy	threat	konvooi	plezier

*salt*	gosh	*zout*	deel

*bowl*	tune	*schaal*	wonder

*bean*	loud	*boon*	stil

*toes*	firm	*tenen*	smaak

*leaf*	host	*blad*	keus

*disc*	meal	*schijf*	waarde

*tear*	posh	*traan*	beeld

*seed*	load	*zaad*	plek

*plum*	hero	*pruim*	geval

*rice*	mood	*rijst*	bevel

*teeth*	proof	*tanden*	risico

*honey*	stuff	*honing*	weldra

*diary*	sweet	*dagboek*	cultuur

*brush*	space	*borstel*	waanzin

*spoon*	death	*lepel*	vraag

*shell*	front	*schelp*	aardig

*snail*	order	*slak*	stem

*candy*	issue	*snoep*	macht

*straw*	civil	*rietje*	afloop

*thumb*	sense	*duim*	raar

*cheese*	amount	*kaas*	moed

*bottle*	injury	*fles*	maat

*rabbit*	scheme	*konijn*	geloof

*button*	relief	*knop*	paar

*salmon*	member	*zalm*	vrij

*throat*	favour	*keel*	zorg

*cotton*	appeal	*katoen*	schuld

*pebble*	action	*kiezel*	proost

*carrot*	genius	*wortel*	ingang

*ribbon*	wealth	*lint*	gauw
